# Charge-Convertible and Reduction-Sensitive Cholesterol-Containing Amphiphilic Copolymers for Improved Doxorubicin Delivery

**DOI:** 10.3390/ma15186476

**Published:** 2022-09-18

**Authors:** Zhao Wang, Xinyu Guo, Lingyun Hao, Xiaojuan Zhang, Qing Lin, Ruilong Sheng

**Affiliations:** 1 School of Material Engineering, Jinling Institute of Technology, Nanjing 211169, China; 2 Nanjing Key Laboratory of Optometric Materials and Technology, Nanjing 211169, China; 3 CQM-Centro de Quimica da Madeira, Campus da Penteada, Universidade da Madeira, 9000390 Funchal, Madeira, Portugal

**Keywords:** charge-convertible, reduction-sensitive, drug delivery, cholesterol

## Abstract

For achieving successful chemotherapy against cancer, designing biocompatible drug delivery systems (DDSs) with long circulation times, high cellular endocytosis efficiency, and targeted drug release is of upmost importance. Herein, a well-defined PEG-*b*-P(MASSChol-*co*-MANBoc) block copolymer bearing redox-sensitive cholesteryl-side group was prepared via reversible addition-fragmentation chain transfer (RAFT) polymerization (with non-redox PEG-*b*-P(MACCChol-*co*-MAN-DCA) as the reference), and 1,2-dicarboxylic-cyclohexene acid (DCA) was then grafted onto the hydrophobic block to endow it with charge-convertible characteristics under a tumor microenvironment. The amphiphilic copolymer could be assembled into polymeric spherical micelles (SSMCs) with polyethylene glycol (PEG) as the corona/shell, and anti-cancer drug doxorubicin (DOX) was successfully encapsulated into the micellar core via strong hydrophobic and electrostatic interactions. This nanocarrier showed high stability in the physiological environment and demonstrated “smart” surface charge conversion from negative to positive in the slightly acidic environment of tumor tissues (pH 6.5~6.8), as determined by dynamic light scattering (DLS). Moreover, the cleavage of a disulfide bond linking the cholesterol grafts under an intracellular redox environment (10 mM GSH) resulted in micellar dissociation and accelerated drug release, with the non-redox-responsive micelles (CCMCs) as the control. Additionally, a cellular endocytosis and tumor proliferation inhibition study against MCF-7 tumor cells demonstrated the enhanced endocytosis and tumor cell inhibitory efficiency of dual-responsive SSMCs/DOX nanomedicines, revealing potentials as multifunctional nanoplatforms for effective oncology treatment.

## 1. Introduction

In recent years, cancer has become one of the leading causes of death for humankind. Tremendous efforts have been devoted to developing efficient cancer treatment strategies, especially chemotherapy [1]. However, traditional chemotherapy still exhibits limited therapeutic efficacy and multiple side effects due to non-specific drug delivery [2,3]. Many researchers have made great advances in developing drug delivery systems (DDSs) to improve therapeutic effects by optimizing targeted drug delivery [4,5,6]. Among these, polymeric nanosystems, including nanoparticles (NPs), micelles (MCs), liposomes, and nanogels, have come to be seen as the most interesting DDSs due to their flexibility in regulating chemical structures and functions, such as stimulus responsibility, controlled drug release, stealth properties, enhanced endocytosis via ligand-mediated active targeting, etc. [7,8,9,10,11].

As effective DDSs, desired polymeric micelles should escape from rapid renal clearance by the reticuloendothelial system (RES), maintain stability and longevity in the bloodstream, deliver cargo to the tumor site, and release drugs efficiently to the targeted location. Recently, some “smart” nanoplatforms, which can respond to the tumor microenvironment (TME) (e.g., pH, hypoxia, redox condition, overexpressed enzymes) or physical stimulus factors (e.g., heat, light, ultrasound, magnetism), have been designed [12,13]. Particularly, TME became a preferable tumor target in theragnostics with the advantages of stable and robust characteristics [14]. Similarly, the extracellular pH (pHe) in solid tumors (6.5~6.8) is lower than that of normal physiological pH (7.2~7.4), and tends to be more acidic in the intracellular endo-/lysosome (pH~5.0) [15]. In addition, the remarkable cytosolic reduction potential with concentrated glutathione (GSH) of 1~10 mM is around 100~1000 times higher than that of extracellular fluids (2~10 µM), and the extracellular GSH concentrations in cancer cells are at least four times higher than those in normal tissues [16]. Thus, taking advantage of the pH and/or GSH concentration gradient in the TME to introduce stimuli-responsive linkers into the polymeric vectors provides a versatile strategy for enhancing cellular endocytosis and improving therapeutic effects [17,18,19,20].

The stability and cellular endocytosis efficiency of NPs greatly rely on their physicochemical properties (e.g., surface charge [21,22,23]). It has been reported that cationic NPs can strongly interact with negatively charged cell membranes by electrostatic forces, thus facilitating rapid cellular internalization [24]. However, the NPs with positively charged surfaces may cause non-specific interactions with the serum proteins, which are easily recognized by the RES and quickly cleared from the blood circulatory system [25], diminishing their in vivo bioavailability. Recently, pH-triggered charge-convertible polymeric NPs have obtained significant attention in solving the dilemma between prolonged blood circulation time and enhanced tumor affinity [26,27,28,29]. Kataoka et al. [30] first reported the concept of citraconic amide-based charge-convertible polymers, which were stable under neutral/basic conditions but degraded under a slightly acidic tumor microenvironment, leading to the transformation of negatively charged carboxylate into positively charged primary amines. Thereafter, maleic acid derivatives such as 2,3-dimethylmaleic acid (DMMA) [31,32,33,34], citraconic acid [35], or 1,2-dicarboxylic-cyclohexene acid (DCA) [36,37,38] were connected onto amides to construct charge-convertible polymeric vectors. Similarly, Chen et al. [39] developed shell-stacked nanostructures based on DMMA-modified polypeptide through electrostatic interactions. These could undergo prominent acid-induced surface charge conversion, and enhance endocytosis and tumor penetration in deep acidic tumor tissue. Zhang et al. [36] prepared acid-labile β-carboxylic amide (C16N-DCA) amphiphile with an alkyl chain and anionic headgroup. Under a tumor acidic microenvironment, the negative–positive charge conversion of the headgroup facilitated significant plasma membrane disruption-induced tumor cell inhibition.

On the other hand, as a natural steroidal compound with a rod-like mesogenic unit [40], cholesterol has been welcomed as a biocompatible hydrophobic moiety with which to construct functional nanocarriers with enhanced stability [41]. Cholesterol interacts with anti-cancer drugs such as paclitaxel (PTX) or doxorubicin (DOX) through strong hydrophobic interactions, thus greatly enhancing their drug loading capabilities [42,43]. As a part of mammalian cell membranes, cholesterol plays an essential role in NPs’ intracellular trafficking through cell surface-recognition and NPC1L1/ABCA1-dependent cholesterol transportation [44], which makes cholesterol-based amphiphiles a good candidate in nanobiomedicine [45,46,47,48]. However, the highly ordered liquid crystalline (LC) phase structure and the strong hydrophobic interactions impede the release of loaded drugs and their practical applications [49]. Liu et al. [50] designed an amphiphilic polysialic acid (PSA) cholesterol derivative with dual-responsive features, in which the cholesterol moiety grafted onto a PSA skeleton via a GSH-responsive disulfide bond to realize efficient drug release in the target tissue. Our previous research on diblock glycopolymer PMAgala-*b*-P(MAA-*co*-MAChol) amphiphiles revealed that the random incorporation of methacrylic acid (MAA) into a cholesterol hydrophobic block could effectively decrease the formation of a condensed LC phase and thus benefit controlled drug release [51]. Thus, the random introduction of the second chemical component in a cholesterol hydrophobic block or constructing a cholesterol-pending block with cleavage linkers to achieve controlled drug release is a promising method for the design of cholesterol-based drug carriers [52].

**Scheme 1:**
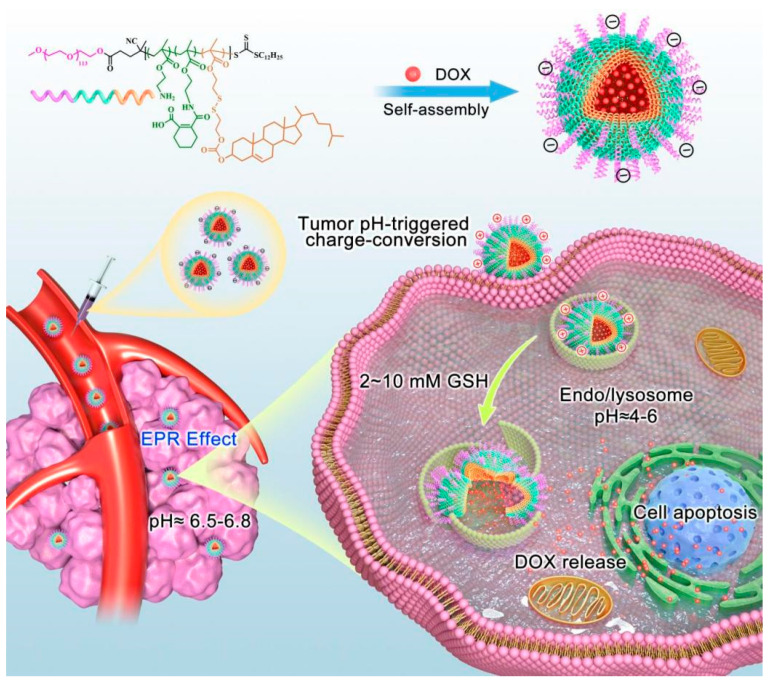
Schematic illustration of amphiphilic copolymer carriers with tumor pH-triggered charge-convertible and reduction-responsive drug release characteristics for prolonged blood circulation and accelerated extracellular doxorubicin release.

Herein, we developed a dual-responsive (pH/reduction) charge-convertible block copolymer PEG-*b*-P(MASSChol-*co*-MANBoc) with cholesterol grafts for efficient DOX delivery; a non-redox PEG-*b*-P(MACCChol-*co*-MAN-DCA) was synthesized as the reference. In our design, hydrophilic poly (ethylene glycol) (PEG) was utilized to facilitate the formation of a “shielding corona” outside the micelles to protect the system from RES clearance for a prolonged body fluid circulation time, which may improve nanomedicine enrichment at the tumor region via the EPR effect before intratumor charge-conversion response. The hydrophobic DCA-containing block was randomly copolymerized with a cholesterol-containing block to enhance the micellar stability and drug loading capability. Once in acidic tumor tissues, the pH-labile β-carboxylic amide bond could be readily cleaved. The resulting hydrophilic 2-aminoethyl methacrylate (MAN) with a high positive zeta potential could enhance cellular uptake. Moreover, the cholesterol was grafted onto the backbone via redox-cleavable disulfide (-SS-) linkage. After being internalized into the cells, the disulfide bond could be cleaved in response to high cellular GSH concentrations. The disruption of hydrophilic–hydrophobic balance results in micellar dissociation and drug release. Furthermore, the random copolymerization of the hydrophilic MAN segment with a cholesterol-containing block break downs the LC phase structure and cholesterol hydrophobic microdomain, contributing to accelerated drug release (Scheme 1). In this study, the structure of a well-designed amphiphilic block copolymer was characterized, and the stimuli-responsiveness and controlled drug release were investigated to prove our design methodology.

## 2. Materials and Methods

### 2.1. Materials

Cholesteryl chloroformate (98%), 2-hydroxyethyl disulfide (90%), and 1,6-hexanediol (98%) were supplied by Acros. 3,4,5,6-Tetrahydrophthalic anhydride (TDA, 98%) was purchased from Energy Chemical. Methacryloyl chloride (98%), 1,8-diazabicyclo [5.4.0]undec-7-ene (DBU, 98%), N,N′-dicyclohexylcarbodiimide (DCC, 98%), 4-(dimethylamino) pyridine (DMAP, 98%), and sodium dodecyl sulfate (SDS, 99%) were supplied by Shanghai Sinopharm Chemical Reagent Co., Ltd. (Shanghai, China). Poly(ethylene glycol) (PEG 5K) and glutathione (GSH, 98%) were supplied by Sigma & Aldrich (Burlington, MA, USA). N,N′-azobis (isobutyronitrile) (AIBN, 98%, J & K, Beijing, China) was recrystallized from methanol prior to use. 4-Cyano-4-(dodecylsulfanyl thiocarbonyl) sulfany pentanoic acid (CDP) was prepared as described in the literature [53]. Toluene was refluxed over metallic sodium and distilled before use. In addition, a cellulose dialysis membrane with a molecular weight cut-off of 3500 was purchased from Shanghai Yuanye Science & Technology Development Co., Ltd. (Shanghai, China). Doxorubicin hydrochloride (DOX·HCl, 98%) was supplied by Dalian Meilun Biotech Co., Ltd. (Dalian, China). Fetal bovine serum (FBS, Cat#10099-141), penicillin-streptomycin solution (Cat#MG7989), and bovine serum albumin (BSA, Cat#0332) were purchased from Genebase Gene Tech Co., Ltd. (Shanghai, China), Invitrogen (Waltham, MA, USA), and Amresco (Solon, OH, USA), respectively. Hoechst 33342 (Cat#MG1790), Lysotracker Green (Cat#MF8124G), and Cell counting kit-8 (CCK-8, Cat#MG6432) were purchased from MesGen Biotech. (Shanghai, China).

### 2.2. Characterization

NMR spectra ^1^H NMR spectra were recorded on a Varian-300 FT-NMR spectrometer operating at 300.0 MHz. ^13^C NMR spectra were obtained on a Bruker Avance-400 FT-NMR spectrometer (100.0 MHz for ^13^C nuclei). All of the NMR measurements were conducted at ambient temperature with tetramethylsilane (TMS) as an internal chemical shift reference.

Mass spectra ESI-MS were routinely measured on a Varian SATURN 2000 instrument.

FT-IR spectra FT-IR spectra were obtained on a Bio-Rad FTS-185 spectrometer (Hercules, CA, USA) at room temperature (KBr pellets, 32 scans, spectral range: 4000–500 cm^−1^, resolution: 4.0 cm^−1^).

Gel permeation chromatography (GPC) Molecular weights (*M*_n_, *M*_w_) and molecular weight distributions (PDI, *M*_w_/*M*_n_) of the prepared polymers were characterized on a PerkinElmer 200 GPC at 35 °C (eluent: THF, flowing rate: 1.0 mL/min, GPC traces calibrate: polystyrene standards) (Polymer Laboratories, Church Stretton, UK).

Dynamic light scattering (DLS) Hydrodynamic particle size and surface potential of the nanomicelles were tested on a DLS instrument (Malvern Zetasizer Nano ZS90, Malvern, UK) at 25 °C (laser beam: λ = 633 nm, scattering angle: 90°).

Transmission electronic microscopy (TEM) The micellar solution (1.0 mg/mL) was dropped onto a 300 mesh carbon-coated copper grid, followed by fluid removal using filter paper, before drying in air at room temperature. The morphologies of the micelles were immediately observed on TEM (FEI Tecnai F20, Hillsboro, OR, USA) with an acceleration voltage of 80 kV.

### 2.3. Synthesis

The synthetic routes of monomers, including disulfide-containing methacrylic-cholesterol monomer (MASSChol), non-disulfide-containing methacrylic-cholesterol monomer (MACCChol), and Boc-protected mannose monomer (MANBoc), are shown in Schemes S1 and S2, and the synthetic procedures were described in detail in the Supporting Information S1.

#### 2.3.1. PEG-CDP

In a dry 250 mL flask, PEG-5K (2.5 g, 0.5 mmol), RAFT agent CDP (1.67 g, 4 mmol), DCC (825 mg, 5 mmol), and DMAP (122 mg, 1 mmol) were added into dichloromethane (DCM, 100 mL) solution. Then, the mixture was reacted at room temperature for 48 h. The undissolved materials were removed by filtration, and the reaction medium was vacuum-concentrated and poured into cooled dry diethyl ether (100 mL × 3). The precipitate was collected and dried under vacuum to obtain PEG-CDP.

#### 2.3.2. PEG-*b*-P(MASSChol-*co*-MANBoc)

The block copolymer was synthesized by controlled RAFT polymerization. In brief, MASSChol (318 mg, 0.5 mmol), MANBoc (229 mg, 1mmol), and PEG-CDP (274.5 mg, 0.05 mmol) were placed into a 50 mL Schlenk tube under a nitrogen atmosphere. Then, AIBN (2.5 mg, 0.015 mmol) dissolved in anhydrous toluene (5 mL) was injected into the Schlenk tube and deoxygenated by freeze–pump–thawing cycles (3 times). Thereafter, the tube was put into an oil bath thermostated at 80 °C for 12 h. Afterwards, the polymerization was inhibited by cooling in liquid nitrogen and poured into cooled dry methanol (50 mL × 3). Then, the white precipitates were collected and dried to obtain PEG-*b*-P(MASSChol-*co*-MANBoc).

#### 2.3.3. PEG-*b*-P(MASSChol-*co*-MAN-DCA)

The as-prepared copolymer precursor PEG-*b*-P(MASSChol-*co*-MANBoc) was deprotected in TFA/DCM (1/2, *v*/*v*) at room temperature for 8 h, and the intermediates were obtained by precipitation in cold anhydrous methanol and dried under vacuum. Then, the intermediates were re-dissolved in the DCM solution with DBU (0.2 eq.) and (TDA, 5 eq.) and further reacted at room temperature for 24 h. The products were obtained by precipitation in cooled methanol from the DCM (3 times) and dried under vacuum overnight.

The non-disulfide PEG-*b*-P(MACCChol-*co*-MAN-DCA) diblock copolymers was prepared in a similar manner to the procedure of PEG-*b*-P(MASSChol-*co*-MAN-DCA).

### 2.4. Preparation of Blank Micelles and Their DOX-Loaded Micelles

Blank and DOX-loaded polymer micelles were prepared using a modified nanoprecipitation method. First, PEG-*b*-P(MASSChol-*co*-MAN-DCA) or PEG-*b*-P(MACCChol-*co*-MAN-DCA) block copolymers (10.0 mg) were dissolved in THF (1 mL) and stirred at room temperature for 4 h. Then, phosphate buffer (PBS, pH 7.4, 10 mM, 5 mL) was added dropwise with vigorous stirring. Finally, the mixture was put in preswollen cellulose dialysis tubing (MWCO 3500, Shanghai Green Bird Science & Technology Development Co., Ltd. Shanghai, China) and dialyzed against deionized water for 48 h to obtain the final PEG-*b*-P(MASSChol-*co*-MAN-DCA) micelles (SSMCs) or PEG-*b*-P(MACCChol-*co*-MAN-DCA) micelles (CCMCs). Similarly, DOX (10.0 mg) and 2 molar equiv. triethylamine (TEA) were dissolved into DMSO (1 mL) and stirred for 4 h. Then, the block copolymers (40.0 mg in 4 mL of THF) were added, followed by the dropwise addition of PBS (pH = 7.4, 10 mM, 20 mL). The mixture was then stirred at room temperature for 12 h. Finally, the mixture was put in cellulose dialysis tubing (MWCO 3500) and dialyzed against deionized water for 48 h to give the DOX-loaded micelles (SSMCs/DOX and CCMCs/DOX) solution. The DOX-loaded micelles were lyophilized in the dark, and the DOX amounts were analyzed suing a UV-Vis spectrometer (UV-2800, Hitachi, Tokyo, Japan). Drug loading content (DLC) and drug loading efficiency (DLE) were respectively calculated (with a standard working curve) using the following formulas:

$$\mathrm{DLC}\;\left(\mathrm{wt}\%\right)=\frac{\mathrm{Weight}\;\mathrm{of}\;\mathrm{loaded}\;\mathrm{drug}}{\mathrm{Weight}\;\mathrm{of}\;\mathrm{loaded}\;\mathrm{drug}\;\mathrm{and}\;\mathrm{polymers}}\times 100\%$$

$$\mathrm{DLE}\;\left(\%\right)=\frac{\;\mathrm{Weight}\;\mathrm{of}\;\mathrm{loaded}\;\mathrm{drug}}{\;\mathrm{Weight}\;\mathrm{of}\;\mathrm{drug}\;\mathrm{in}\;\mathrm{feed}}\times 100\%$$

### 2.5. Critical Micelle Concentration (CMC)

CMC values of the as-prepared block copolymers were measured using Pyrene (98%, Sigma Aldrich, St. Louis, MO, USA) as a fluorescence probe. Briefly, pyrene/acetone solution was added into the polymer micelle aqueous solution (the concentration range of 1 × 10^−5^~2 × 10^−1^ mg/mL) to keep the final pyrene concentration of 6.0 × 10^−7^ M. Before measurement, the solution was maintained in the dark for 24 h. The excitation spectra were measured on a fluorescence spectrometer (F-7000, Hitachi, Tokyo, Japan, λ_ex_ = 334 nm, scanning wavelength: 350~450 nm). The ratiometric fluorescence intensity (*I*_394_/*I*_374_) of pyrene probe was calculated and plotted against the micelle concentration to determine the CMC value for each block copolymer [54].

### 2.6. In Vitro DOX Release

DOX release properties were tested under different mediums for the drug-loaded SSMCs and CCMCs at 37 °C. Each DOX-loaded micelles solution (2.0 mg/mL, 2 mL) was put in cellulose dialysis tubing (MWCO 3500) and immersed into a different buffer medium (pH 7.4, 0 mM GSH; pH 6.0, 0 mM GSH; pH 7.4, 10 mM GSH; or pH 6.5, 10 mM GSH) at 37 °C under gentle shaking. At predetermined time intervals, 2 mL of medium was collected, and another 2 mL fresh medium was added for UV-vis analysis. In vitro DOX release profiles were measured on a UV-vis spectrometer according to a standard working curve.

### 2.7. Cell Culture

Human breast cancer cells (MCF-7) were purchased from the Cell Bank of Shanghai Institute of Biochemistry and Cell Biology (Shanghai, China). Cells were cultured in Dulbecco’s modified Eagle’s medium (DMEM) supplemented with 10% (*v*/*v*) FBS, 1 mM sodium pyruvate, 100 IU/mL penicillin, and 100 µg/mL streptomycin under 5% CO_2_ at 37 °C for 24 h until they reached 80% confluence before bioevaluation.

### 2.8. Confocal Laser Scanning Microscopy

MCF-7 cells were seeded into glass-bottom dishes at a density of 1 × 10^6^ cells per well. After 24 h of incubation, the medium was replaced with fresh DMEM medium containing DOX-loaded SSMCs (SSMCs/DOX) or DOX-loaded CCNPs (CCMCs/DOX) with the final DOX concentration of 3 µg/mL. After incubation for 1 h or 4 h, respectively, the culture medium was removed, and the cells were washed twice with PBS. Then, fresh medium containing Hoechst 33342 (final concentration of 2 µg/mL) and Lysotracker Green (3 µg per well) were added and maintained for 10 min. The cells were then washed three times with PBS. Fluorescence images of cells were obtained with a TCS SP5 Confocal Laser Scanning Microscope (Leica, Inc., Thornwood, NY, USA) and analyzed with the Leica 2.6.0 software.

### 2.9. CCK-8 Assay

The cytotoxicity of DOX-loaded micelles in MCF-7 cells was evaluated with an in vitro CCK-8 assay. First, MCF-7 cells were seeded into 96-well microplates (6 × 10^3^ cells/well) in 100 µL of DMEM medium. After 24 h of incubation, the DMEM medium was replaced with a fresh DMEM medium containing SSMCs or CCMCs (final micelle concentration of 0–200 µg/mL) and further incubated for 24 h. Thereafter, CCK-8 solution (10 µL) was added into each well and incubated for 1 h with gentle shaking. Finally, the absorbance was analyzed on a microplate reader (BioTek, ELX800, Winooski, VT, USA) at λ = 490 nm (reference: λ = 630 nm). The relative cell viability (%) was calculated as follows (n = 6):

Cell viability (%) = (OD_490 (sample)_ − OD_490 (blank)_)/(OD_490 (control)_ − OD_490 (blank)_) × 100%

## 3. Results and Discussion

### 3.1. Synthesis and Characterization of PEG-b-P(MAChol-co-MAN-DCA) Block Copolymers

**Scheme 2:**
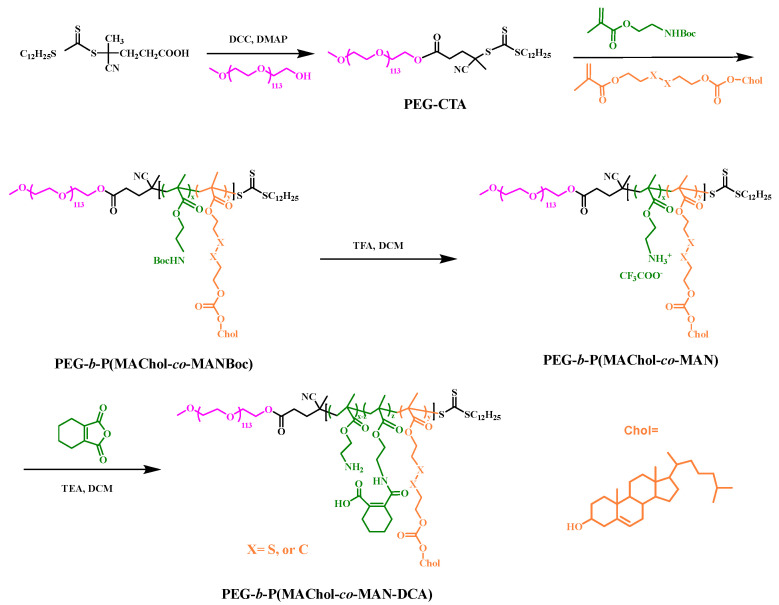
Synthetic route of PEG-*b*-P(MAChol-*co*-MAN-DCA) block copolymers.

**Figure 1:**
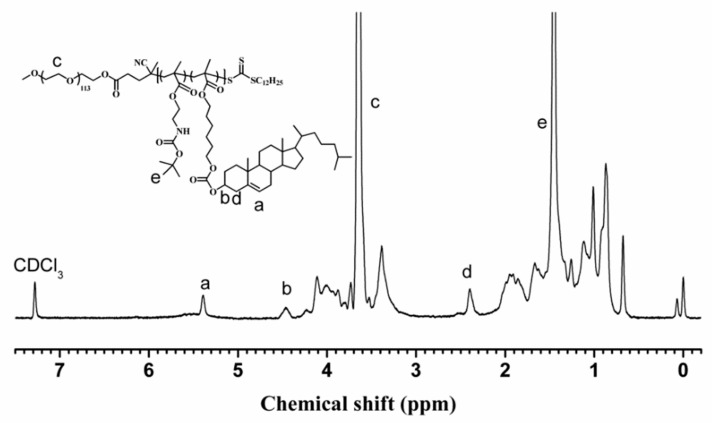
^1^H NMR spectrum for the prepared block copolymer PEG-*b*-P(MACCChol-*co*-MANBoc).

**Figure 2:**
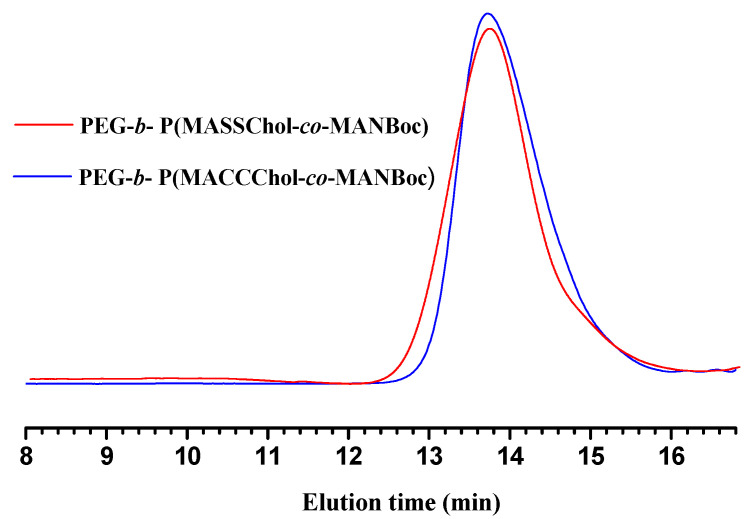
GPC curves for the synthesized PEG-*b*-P(MAChol-*co*-MANBoc) copolymers.

The synthesis strategy of the PEG-*b*-P(MAChol-*co*-MAN-DCA) diblock terpolymers was based on reversible addition-fragmentation chain transfer (RAFT) polymerization and a post-grafting reaction (Scheme 2), since the RAFT polymerization technique could guarantee a controllable molecular weight and low PDI of the synthesized functional copolymers with relatively well-organized structures. First, the monomers MAChol (MACCChol/MASSChol) and MANBoc were synthesized as shown in Schemes S1 and S2. Their ^1^H NMR spectra are depicted in Figures S1 and S2, respectively, and the resonance signals are assigned. Then, the chain transfer agent (CTA) 4-cyano-4-(dodecylsulfanylthiocarbonyl) sulfany pentanoic acid (CDP) was conjugated with PEG-5K with the aid of the coupling agent DCC and DMAP. Diblock terpolymers PEG-*b*-P(MAChol-*co*-MANBoc) were prepared via RAFT polymerization under optimized conditions (toluene, 80 °C) and reagent CTA/MAChol/MANBoc/AIBN was prepared with a molar ratio of 1/10/20/0.3, with PEG-5K as the macro-RAFT agent and MAChol and MANBoc as the comonomer. Accordingly, the degree of polymerization (DP) of MAChol and MANBoc units can be calculated by the monomer conversions based on ^1^H NMR spectra. Figure 1 shows a typical ^1^H NMR spectrum for the resulting PEG-*b*-P(MACCChol-*co*-MANBoc). The characteristic peaks a (5.38 ppm, =C*H*R), b (4.48 ppm, OCOOC*H*R), and d (2.36 ppm, C*H*_2_CHOCOO) belong to the cholesterol segment, peak e (1.45 ppm, (C*H*_3_)CO) belongs to the MANBoc segment, and peak c (3.68 ppm, C*H*_2_C*H*_2_O) belongs to the PEG block, indicating the successful synthesis of the diblock terpolymer structure. GPC curves (Figure 2) and synthetic results for the PEG-*b*-P(MACCChol-*co*-MANBoc) and PEG-*b*-P(MASSChol-*co*-MANBoc) copolymers (Table 1) are presented. The GPC curves exhibited a monodispersed peak with low *M*_w_/*M*_n_ values (PDI ≤ 1.23), substantiating the successful preparation of the copolymer series with a narrow molecular weight distribution. It is worth noting that the synthesized PEG-*b*-P(MACCChol-*co*-MANBoc) and PEG-*b*-P(MASSChol-*co*-MANBoc) showed very similar molecular weights and block weight ratios, which was beneficial for the following comparative study.

**Figure 3:**
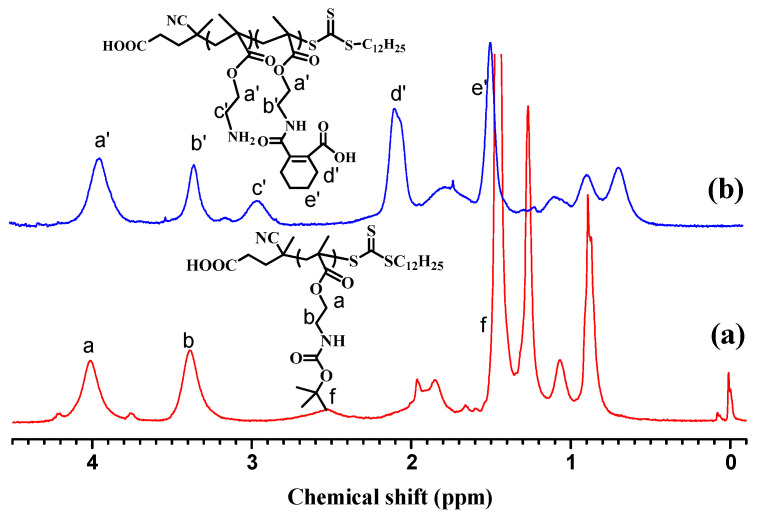
^1^H NMR spectra of the PMANBoc homopolymer in CDCl_3_ (**a**) and P(MAN-DCA) copolymer in D_2_O (**b**).

Then, the protection groups (Boc) of PEG-*b*-P(MACCChol-*co*-MANBoc) and PEG-*b*-P(MASSChol-*co*-MANBoc) were removed with the aid of trifluoroacetic acid (TFA), and the generating amino group was further reacted with 3,4,5,6-tetrahydrophthalic anhydride (TDA) to give the PEG-*b*-P(MAChol-*co*-MAN-DCA) block copolymers. It is a pity that the typical ^1^H NMR signals of MANBoc and DCA moieties overlapped with that of cholesterol skeleton and PEG chains, impeding the observation of the graft reaction by NMR. Hence, a homopolymer PMANBoc and its grafted product P(MAN-DCA) were synthesized (Scheme S3), and ^1^H NMR spectra of the PMANBoc in CDCl_3_ and P(MAN-DCA) in D_2_O are depicted in Figure 3 to provide a deep insight into the grafting reaction. The disappearance of the ^1^H signals (f, 1.45 ppm, (C*H*_3_)CO) for the tert-butyl groups indicated the successful TFA-catalyzed deprotection process. The new signals at δ = 1.53 ppm (e’) and δ = 2.15 ppm (d’) assigned to the DCA skeleton appeared, suggesting the grafting reaction was successfully performed. However, the signal peak at around 2.93 ppm could be identified as the methylene groups adjacent to the free amino group (c’, NH_2_C*H*_2_CH_2_), indicating that the grafting reaction was incomplete due to the steric hindrance of macromolecular reactions. The grafting degree calculated by the integral area ratio of a’, b’, and c’ was 68%.

### 3.2. Preparation and the Dual-Responsiveness of PEG-b-P(MASSChol-co-MAN-DCA) Micelles

**Figure 4:**
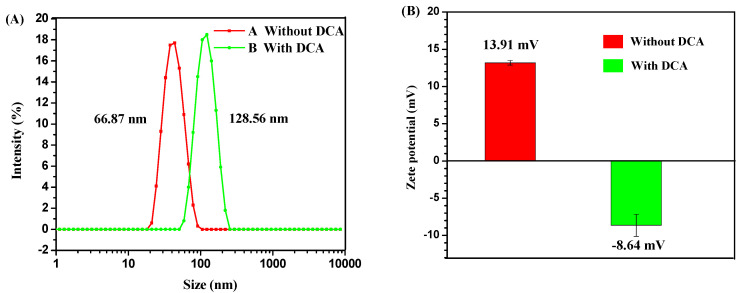
Hydrodynamic particle size (**A**) and zeta potential (**B**) of block copolymers PEG-*b*-P(MASSChol-*co*-MAN-DCA) (with DCA) and PEG-*b*-P(MASSChol-*co*-MAN) (without DCA).

**Figure 5:**
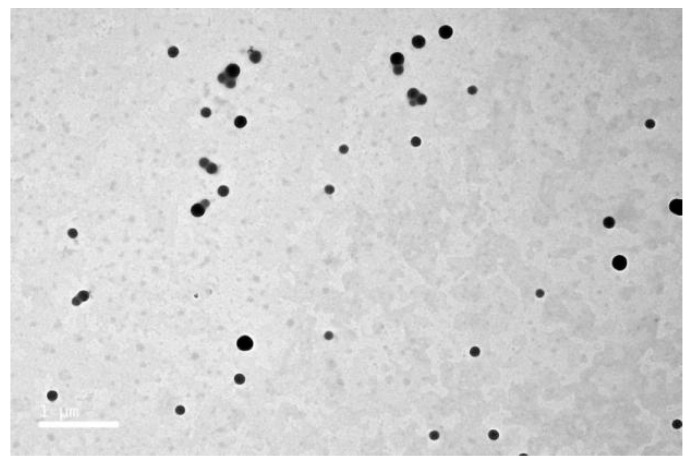
TEM images of SSMCs.

The amphiphilic aggregates of PEG-*b*-P(MASSChol-*co*-MAN) and PEG-*b*-P(MASSChol-*co*-MAN-DCA) were prepared using the nanoprecipitation method. The precursor polymer PEG-*b*-P(MASSChol-*co*-MAN) without DCA grafting was self-assembled into micelles with the size of 66.87 nm and a zeta potential of 13.91 mV, as detected by DLS (Figure 4). After DCA grafting, it exhibited a particle size of 128.56 nm with a narrow size distribution (PDI) of 0.128, and almost monodisperse spherical micelles (SSMCs) could be observed by TEM (Figure 5). The introduction of carboxyl groups and the PEG surface coating on the micelles led to a slightly negative surface potential of −8.64 mV [55]. The increased particle size and zeta potential transformation from positive to negative demonstrated the successful grafting of DCA. The PEG-*b*-P(MACCChol-*co*-MAN-DCA) self-assemblies (CCMCs) showed a similar particle size of 117.6 nm and zeta potential of −7.89 mV, as shown in Table 2. The critical micelle concentration (CMC), an essential parameter for estimating the solution stability of the aggregates, was tested using the fluorescence probe technique. SSMCs and CCMCs showed CMC values of 3.4 mg L^−1^ and 3.8 mg L^−1^ (Table 2). The extremely low CMC values (3.4–3.8 mg/L^−1^) suggested that the PEG-*b*-P(MAChol-*co*-MAN-DCA)-based micelles might possess good post-administration stability, even under extreme dilution conditions within the blood circulation system [56].

**Figure 6:**
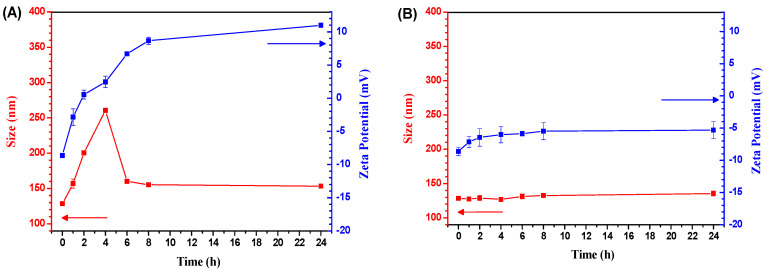
Hydrodynamic particle size and zeta potential of SSMCs after incubation at pH 6.5 (**A**) and pH 7.4 (**B**) in PBS medium for different times.

It was reported that the β-carboxylic amides group was pH-sensitive under weak acidic conditions (pH 6.5–4.5). To investigate whether the prepared PEG-*b*-P(MAChol-*co*-MAN-DCA) micelles showed charge-convertible behavior in the tumoral pH environment, i.e., pH 6.5, the SSMCs were separately incubated under acidic conditions (pH 6.5) and neutral PBS medium (pH 7.4). The sizes and zeta potentials as a function of time were monitored by DLS. As shown in Figure 6, when SSMCs were incubated in a neutral PBS medium (pH 7.4), there was no noticeable size and zeta potential change (Figure 6A). The charge only increased slightly from −8.64 mV to −5.32 mV after 24 h of incubation. However, when the pH reduced (pH 6.5), a significant charge conversion was observed. The zeta potential of SSMCs transformed from -8.64 mV to a positive charge of +2.45 mV after 4 h of incubation and reached +10.98 mV after 24 h of incubation. Moreover, the size of SSMCs rapidly increased to 260.5 nm after 4 h of incubation (Figure 6B). The deposition of the DCA group results in the disruption of hydrophilic/hydrophobic balance, leading to a dissociation-reaggregation process in some micelles. Accordingly, a new equilibrium was established and the average particle size of SSMCs finally stabilized in the solution at ~150 nm after 24 h, which was slightly higher than the original value. Before the charge conversion process, the micelles were progressively circulated in body fluid and enriched/accumulated in the tumor region via the EPR effect. The apparent charge conversion and particle size change demonstrated the hydrolysis of the acid-labile β-carboxylic acid amides group (DCA) under a mildly acidic environment, thus resulting in the exposed amino group. Therefore, the surfaces of the negatively charged micelles could avoid the electrostatic interaction with the negatively charged blood proteins/coagulation factors. Furthermore, the ingenious charge conversion feature produces a strong binding force between the cytomembrane and micelles, enhancing the endocytosis and cellular drug release of the micelles [57].

**Figure 7:**
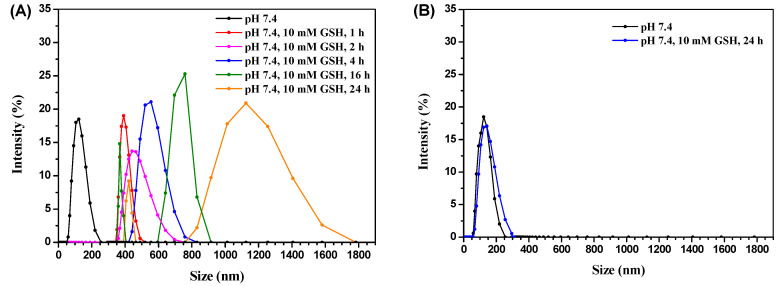
Size distributions of SSMCs (**A**) and CCMCs (**B**) after incubation at pH 7.4 in PBS buffer with 10 mM GSH for different times.

The redox-responsive properties of disulfide-containing SSMCs were investigated by treating SSMCs with 10 mM GSH at 37 °C, and the hydrodynamic particle sizes were measured at predetermined times by DLS. The average particle sizes of SSMCs increased from 128 nm to 427 nm within 1 h and gradually enlarged in 24 h. Double dispersive peaks centered at 350 nm and 780 nm were detected (16 h) and reached 430 nm and 1170 nm after 24 h of incubation, suggesting GSH-induced nanoaggregates/micelles dissociation. In contrast, the particle sizes of the CCMCs determined by DLS saw no noticeable changes with GSH incubation within 24 h (Figure 7B), further demonstrating that the course of the cleavage is dictated by the cleavage of disulfide bonds.

### 3.3. DOX Loading and In Vitro Dual-Responsive DOX Release Behavior

DOX, a widely used clinical anti-cancer drug, was encapsulated into self-assembled micelles via the nanoprecipitation method. The SSMCs and CCMCs showed similar DOX loading properties, with a drug loading efficiency (DLE) of 87.4% and 83.3% (DOX feeding ratio was 20 wt%), and a drug loading content (DLC) of 14.9 wt% and 14.3 wt%, respectively (Table 2). The strong hydrophobic interaction and π-π-stacking between the DOX and cholesterol skeleton gave rise to the high drug loading capacity [48], and the introduction of carboxyl groups further improved the DOX loading capability through electrostatic interactions [58]. The average sizes of the DOX-loaded SSMCs (SSMCs/DOX) and DOX-loaded CCMCs (CCMCs/DOX) were 168.2 nm and 154.3 nm, respectively. The diameter of less than 200 nm might be favorable for tumor vasculature accumulation via the EPR effect.

**Figure 8:**
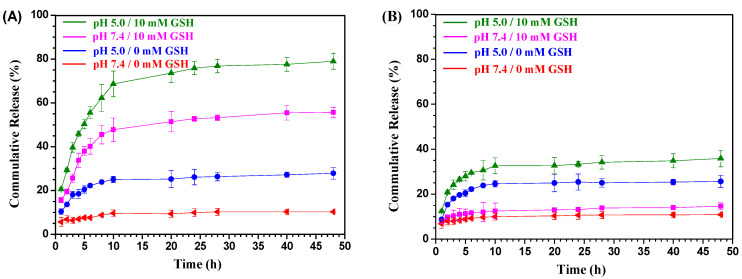
Time-dependent cumulative release of DOX from SSMCs/DOX (**A**) and CCMCs/DOX (**B**) in different incubation mediums.

The in vitro drug release behavior of SSMCs/DOX and CCMCs/DOX was investigated at 37 °C in four different buffer solutions (phosphate buffer solution, pH = 7.4/no GSH; acetate buffer solution, pH = 5.0/no GSH; phosphate buffer solution, pH = 7.4/10 mM GSH; acetate buffer solution, pH = 5.0/10 mM GSH), mimicking the physiological environment and tumor extracellular/intracellular environment. As for the SSMCs/DOX shown in Figure 8A, only about 10% of the drug was released from the micelles after 48 h of incubation under physiological conditions (pH = 7.4/no GSH), indicating excellent stability of the micelles in the bloodstream. However, about 27.8% of the cargo was released in the medium within 48 h when the pH decreased to 5.0, higher than that in the physiological solution. This pH-dependent drug release property was likely caused by the deposition of DCA in the acidic environment, resulting in the enhanced hydrophilicity of the micellar inner core and decreased interaction between the carboxyl groups of DCA and DOX upon protonation at a lower pH [59]. In contrast, at a pH of 7.4, after adding 10 mM GSH, the accumulated DOX release reached 47.8% after 10 h, and 55.7% after 48 h, respectively, attributing to the disulfide bond cleavage-induced dissociation of micelles. Furthermore, accelerated DOX release was detected at a pH of 5.0, and the accumulated DOX release reached 68.7% after 10 h, and 79.0% after 48 h, respectively. The results showed that the DOX releasing from SSMCs proceeds in a controlled manner and could be activated by a synergistic trigger of low pH-redox factors. This was beneficial for enhanced drug release in the cytoplasm and the anti-tumor effect.

For CCMCs/DOX, a similar pH-dependent DOX release manner could also be observed. The accumulated DOX release was 10.9% at pH 7.4 and 25.7% at pH 5.0 within 48 h without GSH. Only a slight difference was observed (3.8% and 10.2%) in cumulative DOX release behavior under the bioreduction environment. This was most likely due to the diffusion of GSH into the micelles. It is worth noting that less than 36.0% of the drug was released after incubation at pH 5.0 and 10 mM GSH for 48 h for CCMCs, which is much lower than the 79.0% for the SSMCs under otherwise similar conditions. The data further demonstrated that the accelerated DOX release in a reducing environment was due to the disassembly of self-assembled micelles coming from the cleavage of disulfide leakage.

### 3.4. Dual-Responsive SSMCs Enhance the Intracellular Release of DOX

**Figure 9:**
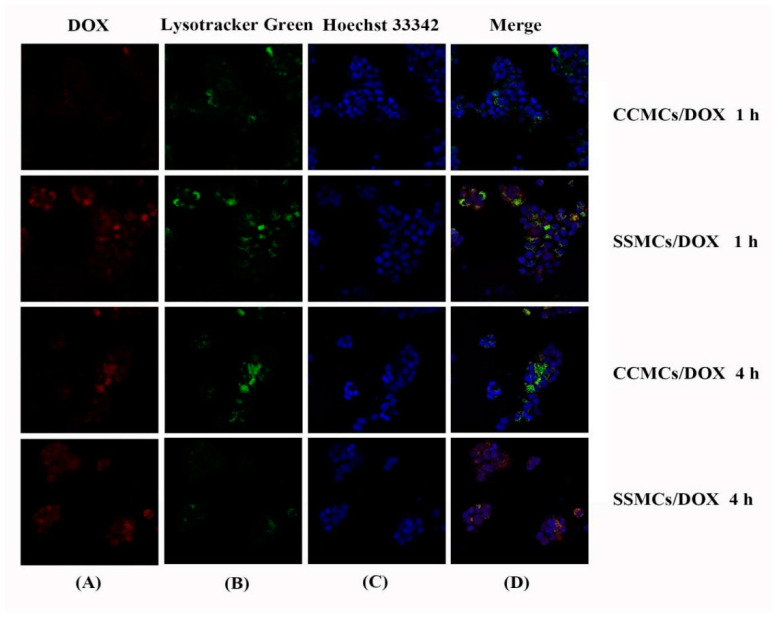
CLSM images of MCF-7 cells following 1 h and 4 h fo incubation with CCMCs/DOX and SSMCs/DOX. For each panel, the images from left to right showed (**A**) DOX fluorescence in cells (red); (**B**) lyso-endosomes stained by Lysotracker Green (green); (**C**) cell nuclei stained by Hoechst 33342 (blue); and (**D**) overlays of three images.

Confocal fluorescent microscopy was further used to investigate the transport behavior and intracellular DOX release of DOX-loaded micelles. MCF-7 cells were cultured with SSMCs/DOX or CCMCs/DOX for 1 h and 4 h at 37 °C, respectively. The cell nucleus stained with Hoechst 33342 was in blue, and endosome/lysosome stained with Lysotracker Green was in green. As shown in Figure 9, for SSMCs/DOX, the red fluorescence of DOX can be observed in the cytoplasm after 1 h of incubation, demonstrating its rapid cellular endocytosis. Moreover, most of the DOX red fluorescence was observed in the perinuclear region of cells, indicating that the nanosystems were mainly located in the cytoplasm during the initial stage. A larger amount of DOX (red fluorescence) overlapped with the cell nuclei (blue fluorescence) when the incubation time was prolonged to 4 h. The data revealed the remarkable endosomal escape capability, accompanied by redox-sensitive disulfide bond cleavages and controlled drug release. This is because the SSMCs/DOX with an average particle size of ~156 nm cannot cross the nuclear pores (<39 nm) and enter the nucleus directly [60]. In contrast, it shows weak fluorescence signals from CCMCs/DOX (reduction-insensitive control) after incubation for 1 h, and very weak DOX fluorescence was observed in the perinuclear region of cells after incubation for 4 h under otherwise similar conditions. The results indicated that pH-redox dual-responsive SSMCs could lead to higher efficient intracellular DOX release than the non-redox CCMCs counterpart. The different intracellular drug release behaviors might be due to the fast and efficient DOX release from SSMCs following their rapid cellular internalization and bioreduction-triggered dissociation in the cytoplasm, which is in accordance with the in vitro DOX release behaviors.

### 3.5. In Vitro Cytotoxicity and Tumor Cell Proliferation Inhibition

**Figure 10:**
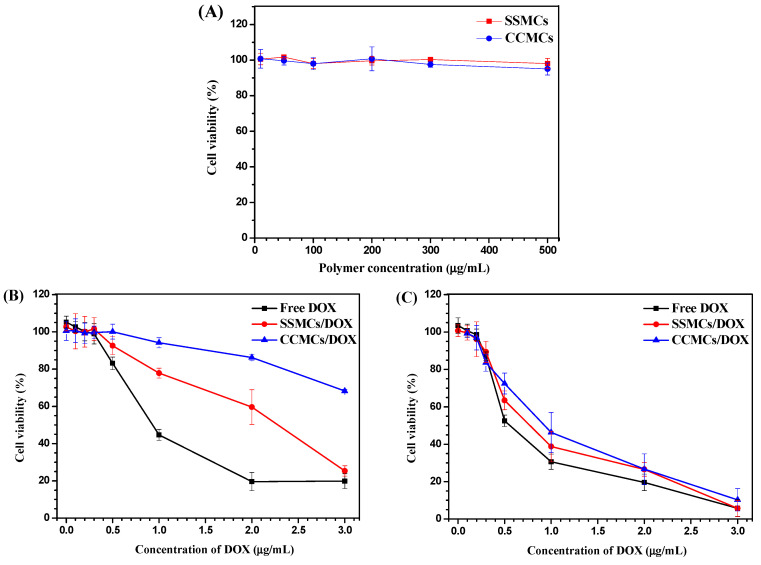
In vitro cytotoxicity of SSMCs and CCMCs to MCF-7 cells (**A**); in vitro cytotoxicity of SSMCs/DOX, CCMCs/DOX, and free DOX determined by the CCk-8 assay at various DOX concentrations for 24 h (**B**) and 48 h (**C**).

Firstly, cytotoxicity of the as-prepared SSMCs and CCMCs was evaluated by CCK-8 assays in MCF-7 cell line. As shown in Figure 10A, both SSMCs and CCMCs had a negligible in vitro impairment in cell viability even with an administration dosage up to 500 µg/mL (cell viability > 95%) after 24 h of incubation, indicating its good cytocompatibility and suitability as a drug carrier in tumor therapy.

Thereafter, time- and dose-dependent tumor cell proliferation inhibition studies were undertaken to determine the effect of DOX delivery. The cytotoxicity of the three DOX formulations (free DOX, SSMCs/DOX, and CCMCs/DOX) were found to be both time- and dose-dependent. After 24 h of incubation (Figure 10B), SSMCs/DOX demonstrated efficient inhibition in cell growth so that only 25% of the MCF-7 cells remained viable at a DOX dosage of 3 µg/mL, similar to that of free DOX (20%). The CCMCs/DOX showed significantly reduced toxicity as compared with SSMCs/DOX, with the DOX concentration varying from 0.5~3 µg/mL, and 68% of the MCF-7 cells remaining viable at a DOX dosage of 3 µg/mL. When the incubation time was prolonged to 48 h (Figure 10C), it could be seen that the gap in cell viabilities at the same DOX concentrations between the three DOX formulations became smaller. The half-maximal inhibitory concentration (IC_50_) values calculated using the GraphPad Prism software of free DOX, SSMCs/DOX, and CCMCs/DOX were 0.50 µg/mL, 0.72 µg/mL, and 1.06 µg/mL, respectively. The difference in the tumor cell proliferation inhibition capability between the three DOX formulations was derived from the endocytosis pathway and DOX release behavior. DOX could rapidly penetrate through the cell membrane via passive diffusion and fast transport to the cell nucleus [61]. SSMCs/DOX with a diameter of less than 200 nm and a cleavage disulfide bond facilitate efficient cellular uptake and drug release to achieve a remarkable triggered cell killing activity as compared with the non-responsive CCMCs/DOX control. These results are consistent with the in vitro and intracellular DOX release behavior shown in Figure 8 and Figure 9.

## 4. Conclusions

In summary, we successfully developed a dual-sensitive (pH/reduction) charge-convertible block copolymer with cholesterol grafts for efficient DOX delivery. The structural and physical characterization demonstrated the controlled RAFT polymerization of the PEG-*b*-P(MASSChol-*co*-MANBoc) precursor. The DCA grafting endows the diblock copolymer with negative surface charge. DLS assays demonstrated that the surface charge of the micelles is converted from negative to positive under a weak acidic tumor microenvironment. Moreover, micelles dissociate under intracellular reduction conditions (GSH concentration of 10 mM). In vitro DOX release profiles demonstrated the high stability of the DOX-loaded SSMCs under neutral conditions, and significantly fast responsive DOX release under acidic and bioreduction environments, with a cumulative release ratio of up to 79% within 48 h. The tumor cell proliferation inhibition study and intracellular localization assay further disclosed the enhanced DOX release and anti-tumor efficacy of SSMCs/DOX, compared with the non-redox-responsive CCMCs/DOX counterpart. In general, introducing a charge-convertible block into a disulfide-linking/cholesterol-grafting block provides a strategy for solving the limitations related to a short blood circulation time, low drug loading efficiency, and controlled intracellular drug release, which paves the way for new anti-tumor drug delivery systems. Additionally, in our subsequent research, we will further compare in vitro/vivo drug targeting and chemotherapeutic effects in normal and tumor cells/tissues.

## Figures and Tables

**Table 1:** Synthetic results for the as-prepared PEG-*b*-P(MAChol-*co*-MANBoc) diblock copolymers.

Sample ^a^	MAChol Conver. (%) ^b^	MApEA Conver. (%) ^b^	*M*_n_, _thero_^c^ (×10^4^)	*M*_w_, _expt_^d^ (×10^4^)	*M*_n_, _expt_^d^ (×10^4^)	*M*_w_/*M*_n_^d^
PEG_113_-*b*-P(MACCChol_8_-*co*-MANBoc_18_)	82	89	1.44	1.86	1.38	1.23
PEG_113_-*b*-P(MASSChol_8_-*co*-MANBoc_17_)	77	87	1.43	1.82	1.41	1.21

Notes: ^a^ Degrees of polymerization (DP) for the PMAChol and PMANBoc blocks were estimated based on monomer conversions by ^1^H NMR. ^b^ Initial PEG-CTA/MAChol/MANBoc/AIBN feeding molar ratio of RAFT polymerization was set to be 1/10/20/0.3. The monomer conversions were calculated by ^1^H NMR. ^c^ Theoretical molar mass *M*_n_ were calculated based on monomer conversions. ^d^ Number-average molar mass (*M*_n_), weight-average molar mass (*M*_w_), and polydispersity index (*M*_w_*/M*_n_) were measured by GPC using polystyrene (PS) for standard calibration.

**Table 2:** Particle sizes and DOX loading behavior of SSMCs and CCMCs.

Sample	Blank Micelles				DOX-Loaded Micelles			
	Size ^a^ (nm)	PDI ^a^	Zeta Potential ^a^ (mV)	CMC ^b^ (mg L^−1^)	Size ^a^ (nm)	PDI ^a^	DLC (wt%) ^c^	DLE (%) ^c^
SSMCs	128.6	0.128	−8.64	3.8	168.2	0.178	14.9	87.4
CCMCs	117.6	0.169	−7.89	3.4	154.3	0.206	14.3	83.3

Notes: ^a^ The size, PDI, and zeta potential were determined by DLS (n = 3). ^b^ Critical micelle concentration (CMC) values were measured using a fluorescence spectrometer with pyrene as the probe. ^c^ The drug loading content (DLC) and drug loading efficiency (DLE) were measured with initial drug feeding weight ratio of 20%.

## Data Availability

The data presented in this study are available upon request from the corresponding author.

## Supplementary Materials

The following supporting information can be downloaded at: https://www.mdpi.com/article/10.3390/ma15186476/s1. Figure S1: ^1^H NMR spectrum of the MACCChol and MASSChol monomers in CDCl_3_; Figure S2: ^1^H NMR spectrum of the MANBoc monomer in CDCl_3_; Scheme S1: Synthetic route of MASSChol monomer; Scheme S2: Synthetic route of MANBoc monomer; Scheme S3: Synthetic route of PMANBoc and P(MAN-DCA).
