# Supplementary material for: Charge-Convertible and Reduction-Sensitive Cholesterol-Containing Amphiphilic Copolymers for Improved Doxorubicin Delivery
Source: Materials (Basel). 2022 Sep 18;15(18):6476. doi: 10.3390/ma15186476 (PMC9504105; doi:10.3390/ma15186476)
Supplement: Supplementary file 1 [file materials-15-06476-s001.zip › materials-1838668-supplementary.pdf]

Supporting Information

Charge-Convertible and Reduction Sensitive Cholesterol-containing Amphiphilic Copolymers for Improved Doxorubicin Delivery

Zhao Wang[1,2], Xinyu Guo[1,2], Lingyun Hao[1,2], Xiaojuan Zhang[1,2], Qing Lin[1,2] and Ruilong Sheng[3*]

1. *School of Material Engineering, Jinling Institute of Technology, Nanjing, 211169, China*
2. *Nanjing Key Laboratory of Optometric Materials and Technology, Nanjing, 211169, China*
3. *CQM-Centro de Quimica da Madeira, Universidade da Madeira, Campus da Penteada, 9000390, Funchal, Madeira, Portugal*

Correspondence: wangzhao@jit.edu.cn; ruilong.sheng@staff.uma.pt; Tel: +351-291705254

S1 Preparation of MASSChol and MACCChol monomers

MASSChol monomer was synthesized by two steps as shown in Scheme S1. Briefly, 2-Hydroxyethyl disulfide (9.24 g, 15 mmol), triethylamine (TEA, 3.41 g, 34 mmol) and dichloromethane (DCM) (100 mL) were added into a 200 mL dry flask. Cholesteryl chloroformate (5.84 g, 13 mmol) dissolved in DCM (50 mL) was added dropwise into the reaction flask under the ice bath. The mixture was stirred at room temperature for 1 h. After removal of the solvent, the crude product was purified by silica gel column chromatography (eluent: DCM) to give the white solid product HO-SS-Chol (3.61 g, 49%). Then, HO-SS-Chol (0.85 g, 1.5 mmol), TEA (0.31 g, 3.0 mmol) and DCM (30 mL) were added into a 100 mL dry flask, and placed in an ice bath. After purging the vessel with N_2, methacryloyl chloride (0.31 g, 3.0 mmol) dissolved in DCM (20 mL) was added dropwise into the mixture, and kept stirring for 20 min. The mixture was naturally warming to room temperature and stirred for another 4 h, then added methanol (10 mL) to quench the reaction. After removal of the solvent, the crude product

was purified by silica gel column chromatography (eluent: DCM/hexane=1:2) to give the product as a white solid (0.33 g, 86%).

^{1}H NMR ($CDCl_3$, δ in ppm): 6.20 (m,1H, =C*H*H), 5.65 (m,1H, =CH*H*), 5.45 (d,1H, =C*H*R), 4.60-4.35(m, 5H, OCOOC*H*R, OCOOC*H*$_2$, COOC*H*$_2$), 3.03(m, 4H, C*H*$_2$SS), 2.50-0.60(m, 46H, other protons on the cholesterol moiety and alkyl chain).

^{13}C NMR ($CDCl_3$, δ in ppm): 154.3, 139.2, 136.0, 126.0, 123.0, 78.1, 65.2, 62.5, 56.6, 42.3, 39.7, 39.5, 38.0, 37.2, 37.1, 36.8, 36.5, 36.1, 35.8, 31.9, 31.8, 28.2, 28.0, 27.6, 24.5, 23.8, 22.8, 22.5, 21.0, 19.2, 18.7, 18.3, 11.8.

FT-IR (in cm^{-1}): 2933.2, 2864.9, 1744.3, 1722.3, 1455.4, 1379.2, 1293.0, 1266.7, 1253.0, 1010.3, 975.9, 944.1, 927.1, 789.9.

ESI-MS [M+Na$^+$] (in m/z): 657.1 (*Calculated* 657.4).

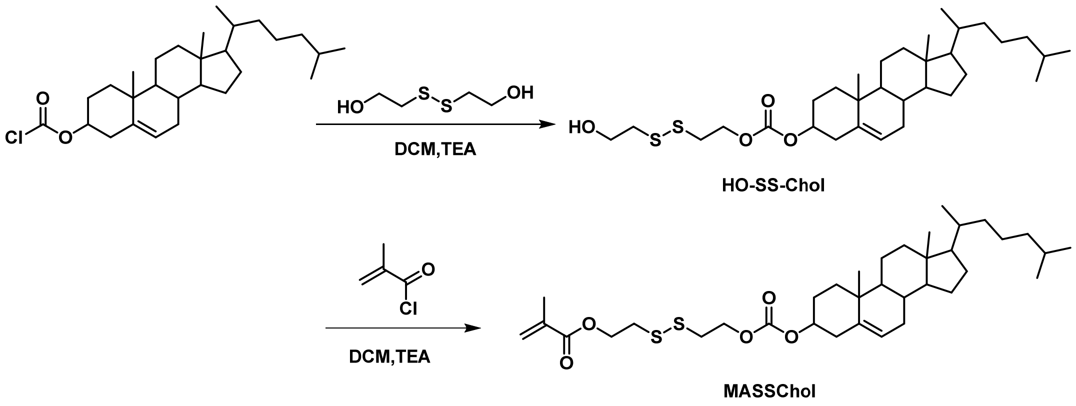

Scheme S1 Synthetic route of MASSChol monomer

MACCChol were prepared similar to the synthetic procedures of MASSChol, in which 1,6-Hexanediol was replaced by 2-Hydroxyethyl disulfide. The total yields of the two steps was 56%.

^{1}H NMR ($CDCl_3$, δ in ppm): 6.10 (m,1H, =C*H*H), 5.58 (m,1H, =CH*H*), 5.40 (d,1H, =C*H*R), 4.48(m, 1H, OCOOC*H*R), 4.20-4.05(m, 4H, COOC*H*$_2$, OCOOC*H*$_2$R), 2.50-0.60(m, 54H, other protons on the cholesterol moiety and alkyl chain).

^{13}C NMR ($CDCl_3$, δ in ppm): 167.5, 154.8, 139.4, 136.4, 125.2, 122.8, 77.7, 67.6, 65.6, 56.7, 56.1, 50.0, 42.3, 39.7, 39.5, 38.0, 36.8, 36.5, 36.2, 37.8, 31.9, 31.8, 28.6, 28.5, 28.2, 28.0, 27.7, 25.7, 25.4,

24.2, 23.8, 22.8, 22.5, 21.0, 19.2, 18.7, 18.3, 11.6.

FT-IR (in cm^{-1}): 2946.3, 2901.7, 2866.6, 1734.6, 1725.4, 1467.6, 1371.3, 1297.1, 1266.7, 1253.6, 1176.5, 991.3, 961.4, 943.9, 793.1.

ESI-MS [$M+Na^+$] (in m/z): 621.3 (*Calculated* 621.5).

S2 Preparation of MANBoc monomers

Ethanolamine (611 mg, 10 mmol), TEA (3.41 g, 34 mmol), Boc_2O (5.84 g, 13 mmol) and DCM (100 mL) were added into anhydrous reaction flask and stirred at room temperature for 12 h. Then, saturated Na_2CO_3 aqueous solution (100 mL) was added into the mixture and stirred for 30 min. Organic phase was separated, and EtOAc (50 mL×3) was used to extract aqueous phase. The combined organic phase was washed three times by dilute hydrochloric acid (pH=3) to remove unreacted raw materials. After dried by anhydrous Na_2SO_4 and removal of the solvent, the intermediate was obtained and utilized for the next steps. Protected ethanolamine (805 g, 5 mmol), triethylamine (1.55 g, 15.0 mmol) and DCM (30 mL) were added into a 100 mL dry flask, and placed in an ice bath. Then, methacryloyl chloride (620 mg, 6.0 mmol) dissolved in DCM (10 mL) was added dropwise into the mixture, and kept stirring for 20 min. The mixture was naturally warming to room temperature and stirred for another 4 h, then added methanol (10 mL) to quench the reaction. After removal of the solvent, the crude product was purified by silica gel column chromatography (eluent: EtOAc/hexane=1:4) to give the product as a white solid. (yield of two steps: 78%).

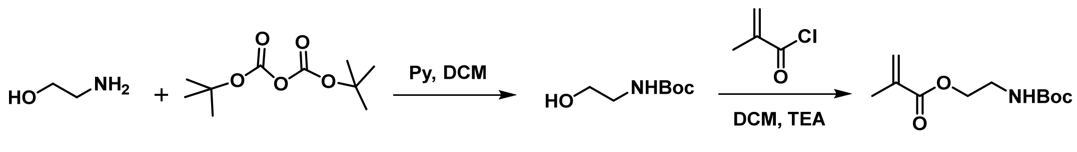

Scheme S2 Synthetic route of MANBoc monomer

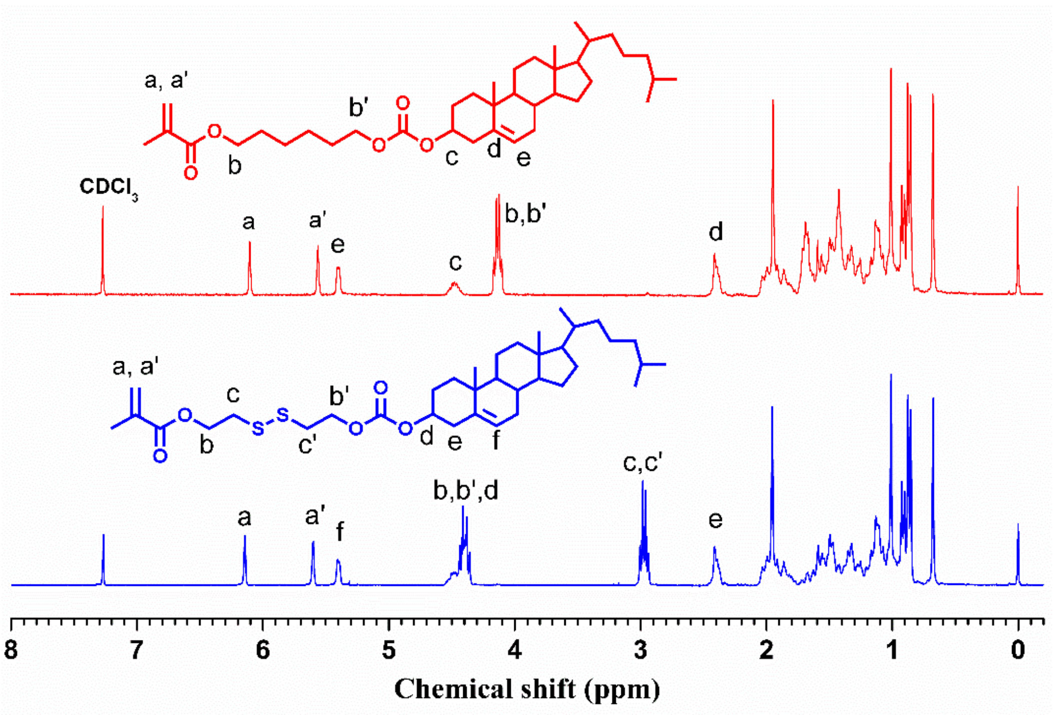

Figure S1. ^{1}H NMR spectrum of the MACCChol and MASSChol monomers in $CDCl_3$

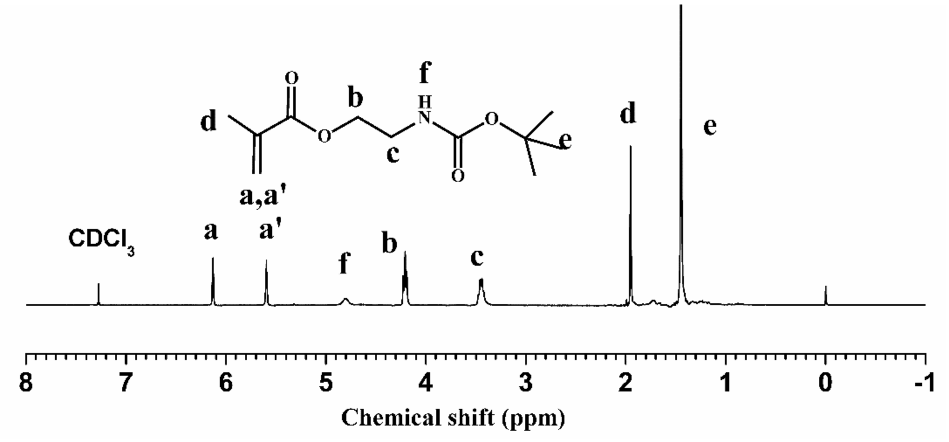

Figure S2. ^{1}H NMR spectrum of the MANBoc monomer in $CDCl_3$

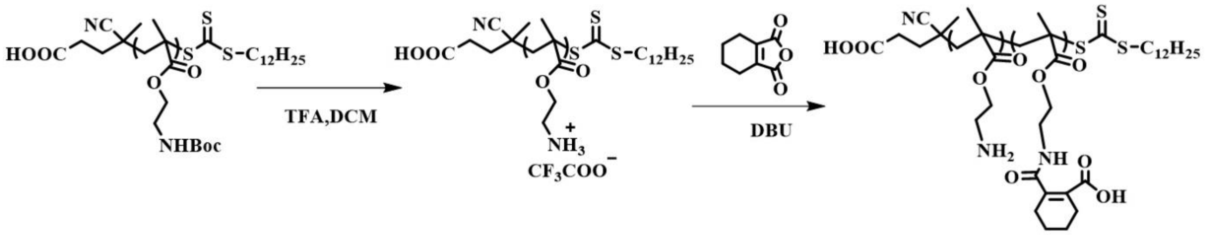

PMANBoc

P(MAN-DCA)

Scheme S3 Synthetic route of PMANBoc and P(MAN-DCA)
